# A Web-Based Intervention Using "Five Ways to Wellbeing" to Promote Well-Being and Mental Health: Randomized Controlled Trial

**DOI:** 10.2196/49050

**Published:** 2024-05-20

**Authors:** Monica Beer Prydz, Nikolai Olavi Czajkowski, Maja Eilertsen, Espen Røysamb, Ragnhild Bang Nes

**Affiliations:** 1 Promenta Research Centre Department of Psychology University of Oslo Oslo Norway; 2 Department of Mental Health and Suicide Norwegian Institute of Public Health Oslo Norway; 3 Department of Childhood and Families Norwegian Institute of Public Health Oslo Norway; 4 Department of Philosophy, Classics, and History of Arts and Ideas University of Oslo Oslo Norway

**Keywords:** well-being, mental health promotion, intervention, web based, low cost, broad outreach, framework, web-based intervention, randomized controlled trial, effectiveness

## Abstract

**Background:**

Compromised well-being and mental health problems pose a significant threat to individuals and societies worldwide. Resource-intensive psychological treatments alone cannot alleviate this burden. There is a need for low-cost, evidence-based interventions aimed at preventing illness and promoting well-being. Five activity domains appear to be linked with well-being promotion across populations: connecting with others, being active, taking notice, learning, and being generous/giving. The activities mentioned are part of the Five Ways to Wellbeing framework and the web-based intervention Five Ways to Wellbeing for All (5waysA).

**Objective:**

This randomized controlled trial aims to test the effects of the 5waysA intervention, a web-based, low-cost, well-being–promoting measure targeting the general population. To date, the Five Ways to Wellbeing framework has not been tested in this specific format. The 5waysA intervention comprises 2 webinars and SMS text message reminders delivered over a 10-week period.

**Methods:**

In 2021, a total of 969 study participants from various regions across Norway were openly recruited through a web page. They were then randomly assigned to either an intervention group or 1 of 2 waiting list control groups, namely, active or passive. Self-reported life satisfaction (Satisfaction With Life Scale [SWLS]), flourishing (Flourishing Scale [FS]), positive emotions, anxiety, and depression symptoms (Hopkins Symptom Checklist-8 [HSCL-8]) were assessed before the intervention, at 4 weeks into the intervention, and 1-2 weeks after the intervention (over 10 weeks). Data analysis was conducted using linear mixed (multilevel) models.

**Results:**

After 10 weeks, 453 participants (171 in the intervention group and 282 in the waiting list control group) were assessed on outcome variables, with a dropout rate of 53.2% (516/969). Results revealed a significantly greater increase in the intervention group compared with the controls for SWLS (b=0.13, 95% CI 0.03-0.23; *P*=.001), FS (b=0.19, 95% CI 0.08-0.30; *P*=.001), positive emotions (b=0.43, 95% CI 0.27-0.60; *P*<.001), and these factors combined into a global well-being measure (b=0.28, CI 0.16-0.39; *P*<.001). Effect sizes (Cohen d) for the well-being outcomes ranged from 0.30 to 0.49. In addition, a significant decrease in anxiety and depressive symptoms was observed (b=–0.17, 95% CI –0.30 to –0.04; *P*=.001) with an effect size (Cohen d) of –0.20.

**Conclusions:**

The findings suggest that the web-based 5waysA intervention could serve as an effective approach for enhancing well-being and mental health within the general population. This study offers individuals, policy makers, and local stakeholders an accessible and potentially cost-effective well-being intervention that could be easily implemented.

**Trial Registration:**

ClinicalTrials.gov NCT04784871; https://clinicaltrials.gov/study/NCT04784871

## Introduction

### Background

The burden on society is significant due to compromised well-being and a sharp increase in mental health issues [1]. Longitudinal studies indicate that more than 80% of the population could meet the diagnostic criteria for a mental disorder at some point in their lifetime [2,3]. The early onset, widespread prevalence, presence of multiple health issues, and prolonged work absences as a result of associated disabilities significantly contribute to the substantial burden of mental health problems and low well-being [4]. Well-being is not simply the absence of mental illness [5]; rather, it encompasses positive elements such as joy and life satisfaction. However, the notably high negative correlation between well-being and mental illness implies a significant connection between them. Thus, gains in levels of well-being can possibly decrease the risk of future mental illness, and vice versa [6,7]. Remarkably, mental illness and compromised well-being can also impact our physical health [8], influencing cardiovascular and immune system functioning [9,10], hastening physical aging [11], and raising mortality rates [12]. Therefore, promoting well-being may enhance not only mental health [1] but also physical health. Evidence-based interventions aimed at promoting well-being, with wide-reaching accessibility and implemented before the onset of mental illness, have been shown to be cost-effective [13,14] and are strongly recommended. In this regard, internet-based interventions emerge as a promising and scalable approach for both treating [15] and preventing [16] mental disorders, as well as enhancing well-being [15,17-19].

Strategies for promoting well-being should target different societal levels and address various risk factors [20]. Poverty and social exclusion are notable risk factors for both compromised well-being and mental health issues, highlighting the necessity of adopting a public health approach and implementing universal interventions, such as political and structural measures [20]. However, the significance of structural strategies aimed at the general population is still not adequately recognized [21], and their implementation within the population is surprisingly limited [22]. In addition to universal interventions, there is a critical need for more targeted interventions that are easily accessible and can be seamlessly integrated into individuals’ daily lives. Recent years have demonstrated that pandemics and related crises can profoundly impact health and well-being on a widespread scale. As a result, the significance of scalable interventions using adaptable delivery methods through technology has become increasingly apparent [23,24].

### Well-Being and Mental Health

Numerous studies using various research designs have consistently demonstrated a close and often prospective association between well-being, mental health, and overall health outcomes, including longevity [8,12,25,26]. The concept and components of well-being have been extensively deliberated upon since ancient times [27]. Contemporary conceptualizations frequently incorporate 3 dimensions of well-being, encompassing how individuals (1) emotionally experience life, (2) cognitively evaluate life, and (3) find meaning and fulfillment in life (eudaimonia) [28,29]. Therefore, well-being entails both feeling good and functioning effectively, including the ability to cope with negative or distressing emotions. In summary, well-being and its components are multidimensional and closely intertwined with mental health, as defined by the World Health Organization (WHO) as “a state of well-being in which individuals realize their own abilities, can cope with the normal stresses of life, can work productively and fruitfully, and are able to make contributions to their communities” [30]. In addition, well-being is closely associated with fundamental psychological needs [31,32].

Well-being is frequently compromised when individuals endure prolonged periods of elevated negative emotions [33]. Similarly, psychopathology is acknowledged as a risk factor for diminished well-being. However, a dual model of mental health underscores that well-being and mental health are distinct, partially independent dimensions. Hence, experiencing high levels of well-being (such as flourishing) does not necessarily imply the absence of mental illness, and conversely, having a mental illness does not mean that joy, meaning, and positive emotions are absent from life [5]. However, the significant correlations between psychopathological symptoms (eg, depression and anxiety) and well-being [6,7] underscore the importance of addressing both well-being and mental health in interventions.

The connections between well-being and both physical and mental health appear to, at least partially, stem from the beneficial and protective effects of positive emotions on health and healthy behaviors [26,34]. Consequently, interventions aimed at promoting well-being and related psychological interventions frequently incorporate the enhancement of positive affect as a target outcome among others.

### The Effectiveness of Interventions

Numerous meta-analyses indicate that various types of psychological interventions can enhance various aspects of well-being while concurrently reducing psychological symptoms, with average Cohen *d* effect sizes ranging between 0.29 and 0.62 [35-38]. Furthermore, lifestyle-oriented interventions that prioritize health behaviors such as physical activity and maintaining a balanced diet have shown promise in promoting well-being and positive mental health [39-41].

Psychological interventions can be costly and resource-intensive, particularly when they require individual face-to-face follow-up. In a health care system under economic strain, there is a need for more efficient solutions. Numerous studies and meta-analyses have shown that web-based interventions are effective alternatives [16,19,24,42-45]. In a recent meta-analysis focusing on positive psychology interventions in nonclinical populations, comparisons were made between interventions using technology-assisted and traditional (ie, face-to-face) methods. The findings indicated that face-to-face interventions were somewhat more effective in enhancing well-being compared with technology-assisted ones (Cohen *d*=0.32 vs 0.17, respectively) [23]. Indeed, research corroborates the effectiveness of stand-alone (ie, without human support) internet and mobile-based psychological interventions in treating various types of psychological issues and improving mental health [16,19,24]. Moreover, web-based psychological interventions have the potential to reach a larger number of individuals in need and are frequently less resource-intensive and costly [15,18]. The ongoing research on the web-based intervention Five Ways to Wellbeing for All (5waysA) seeks to evaluate the effectiveness of a stand-alone, cost-effective, internet-based intervention aimed at promoting well-being with a wide reach.

### The Five Ways to Wellbeing Framework and The Five Ways to All Intervention

#### Overview

The Five Ways to Wellbeing (5ways) originated from a UK government initiative aimed at identifying 5 action domains crucial for promoting well-being and mental health. These action domains were to be evidence based and applicable to individuals of all ages [46]. The resulting 5 action domains are connecting (emphasizing the need for social relations, belongingness, and inclusion), being active (highlighting movement), taking notice (focusing on awareness), keep learning (emphasizing growth, development, and mastery), and giving (encouraging prosocial behavior and adding value). The following sections elaborate on the 5 action domains of the Five ways framework.

#### Connecting

Decades of psychological research have underscored the fundamental human need for attachment [47] and belonging [31,48,49], demonstrating that social relations are indispensable for well-being [50-52]. In a recent study, researchers used network analysis to investigate the connection between well-being and various environmental factors in a sample of 31,000 Norwegian adults. A strong association was observed between perceiving social relations as supportive and rewarding and higher levels of well-being, as well as fewer symptoms of depression and anxiety [53]. In addition, social relations appear to serve as a protective factor against stressors and are linked to mental resilience, even after accounting for genetic factors [54].

#### Be Active

Studies have demonstrated that activity and movement play a crucial role in well-being and can be enhanced through regular practice [41,55,56]. Physical activity has been shown to be effective in alleviating symptoms of depression, anxiety, and psychological distress, both in the general population and in groups with compromised health [57]. Indeed, a recent study using Mendelian randomization provides further evidence supporting a causal relationship between physical activity and the preventative effect on depression [39].

#### Taking Notice

Meta-analyses indicate that engaging in mindfulness practices, which involve being present and aware in the moment, is linked to various benefits for well-being [58-60]. These benefits include changes in brain regions associated with mood modulation [61], suggesting that mindfulness may have a positive impact on mental health. Furthermore, research suggests that awareness, as a skill, can be cultivated and strengthened through practice [32]. In a systematic review and meta-analysis examining the impact of various psychological interventions on well-being, mental, and physical health, mindfulness training and other psychological interventions had the most significant effect on both clinical and nonclinical populations [38].

#### Learning

Humans possess an inherent drive to develop competence, foster curiosity, strive for mastery in diverse life skills, and seek autonomy [62]. Learning and exposure to novelty are linked to the anticipation and sustained engagement of reward circuits in the brain, which typically have positive effects on well-being and serve as a buffer against stress [63,64]. Moreover, introducing novelty into familiar experiences in life appears to be an effective method for enhancing well-being [65,66].

#### Giving

Engaging in acts of kindness typically leads to increased happiness among individuals [67]. Furthermore, being generous and participating in informal giving are associated with various eudaimonic well-being benefits [68] and are considered essential for fulfilling our basic need for relatedness [31]. In addition, the need for mattering, which involves feeling valued and adding value, is emphasized as crucial for a fulfilling life [69]. A recent review of 15 preregistered studies on prosocial spending concluded that such acts not only benefit others but also increase one’s own happiness [70].

Since its launch in 2008, the Five Ways to Wellbeing concept has gained traction in both policy and practice. It has been incorporated into national well-being surveys, school curricula, and local procurement decisions [71]. In 2011, the National Mental Health Development Unit and the National Association of Health Authorities and Trusts Confederation (UK) conducted a survey to assess the extent to which the Five Ways to Wellbeing had been used since its inception. The survey identified 76 instances of the Five Ways to Wellbeing framework being applied across various settings, organizations, and initiatives. However, it also underscored the necessity for rigorous evaluations [71]. Despite the evidence-based nature and widespread adoption of the 5 activity domains, there is a noticeable absence of high-quality preregistered studies demonstrating the effectiveness of these activities when combined. Furthermore, to the best of our knowledge, no one has empirically examined the Five Ways to Wellbeing as a web-based intervention. Research on the Five Ways to Wellbeing framework appears to be limited to a few studies. One previous well-powered cross-sectional study conducted in New Zealand reported positive associations between the 5ways activities and well-being [72]. Similarly, other cross-sectional surveys across different nations have documented a positive relationship [73]. In addition, 2 experimental studies have incorporated the 5ways concept into their research, albeit focusing on different angles [74] and targeting different groups (ie, in-patients) [75] compared with our study.

The web-based 5waysA intervention tested in this study is a multicomponent approach aimed at the nonclinical population. It offers individuals knowledge (well-being literacy), exercises, activities, and experiences tailored to build resources and fulfill basic psychological needs, thereby enhancing well-being. This 10-week intervention is resource effective and delivered via the web, incorporating 2 webinars and subsequent SMS text message reminders to support participants. Adopting a high-quality research design, we used a randomized controlled trial with waitlist controls, encompassing both an active and a passive control group.

## Methods

### Design

This study adheres to a 2-armed randomized controlled trial design and follows the CONSORT (Consolidated Standards of Reporting Trials) guidelines and checklist (Multimedia Appendix 1 [76]) for transparent reporting of clinical trials [77,78]. Initially intending to recruit 1500 participants, recruitment was halted upon reaching a total of 969 participants to align with the project’s time frame. Inclusion criteria were broad, with the only requirement being participants to be 18 years or older, while there were no specific exclusion criteria. All participants were recruited in 3 rounds from across Norway between February 2021 and November 2021. Our primary recruiting channels were the official social media platforms (ie, Facebook and Instagram) of local municipalities, the Norwegian Institute of Public Health, the Norwegian unit of the WHO European Healthy Cities Network, and a women’s magazine (Kamille). The overall recruitment message invited individuals to participate in a study entitled “Hverdagsglede for alle” (Everyday Joy for All) aimed at learning about a health-promoting concept and related exercises to enhance well-being, health, and joy in their everyday lives. Participants were provided with information about the study and the randomization process. We explicitly outlined that 2 groups (the “active” group and the “passive control” group) would have to wait 3-5 months for the intervention, while 1 group (the intervention group) would receive the intervention promptly. Moreover, we openly communicated that 1 of the waiting list groups would receive a small registration exercise while awaiting the intervention. All participants were informed that they were automatically entered into a lottery for 5 gift cards (each worth €50 [US $54]). Data collection was conducted using “Nettskjema,” a Norwegian standard tool for designing and administering online surveys developed by the University Information Technology Center (USIT) at the University of Oslo, Norway. This software is specifically designed for collecting highly sensitive research data, is integrated with the Services for Sensitive Data (TSD), and is known for its user-friendly interface [79].

**Figure 1:**
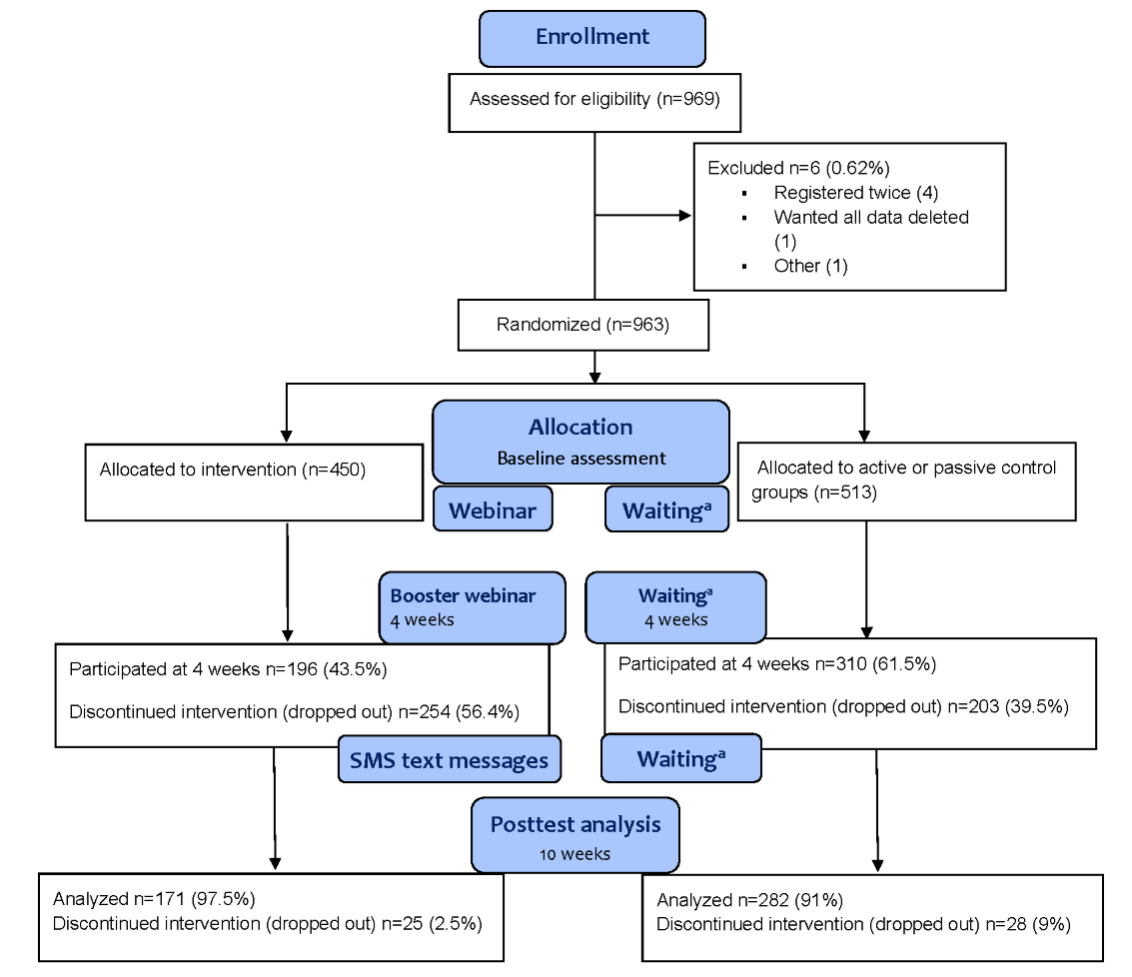
CONSORT (Consolidated Standards of Reporting Trials) flow diagram. ^a^Half of the control group wrote an activity log while waiting.

The randomization was computer generated within the “Nettskjema” solution. As shown in Figure 1, 2 control conditions, namely, the “passive” and “active” control groups, were included to investigate potential differences across control conditions [80] and mitigate the risk of artificially inflating effect sizes [81,82]. The “passive” control group received no recommendations or exercises while waiting. The “active” control group was instructed to use an activity log containing a list of 10 everyday activities. They were asked to indicate whether they had completed these activities or not during the previous week. Participants in the active control group repeated this process for 10 weeks while waiting for the intervention.

### Power

The a priori power analysis was conducted using G*Power3 (The G*Power Team), assuming a small effect size (*f*=0.10) for the intervention, with an α level of .05. Even with a high dropout rate of 50%, our power of 0.999 would still be sufficient to detect a group × time interaction.

### Participant Eligibility

All participants received an online letter of information about the study, which also outlined the requirements of The Regional Committees for Health Research Ethics (REK). Written consent was obtained from all participants in all groups through “Nettskjema,” along with 2-factor authentication (Multimedia Appendix 2). All sensitive personal information was managed in compliance with General Data Protection Regulation (GDPR) requirements. Data were stored and analyzed using the TSD at the University of Oslo.

### Ethics Approval

The trial obtained ethical approval (reference number: 62155) from The Regional Committees for Health Research Ethics (REK).

### The 5waysA Intervention

The 5waysA program is structured as a 10-week intervention comprising a 2-hour webinar, a 1-hour booster webinar (4 weeks later), and 10 SMS text message reminders (Table 1). In summary, the webinars offer a thorough introduction to how stressors in life impact well-being and health. In addition, participants are educated on how the 5ways activities can serve as a remedy for unhealthy stress, elevate positive emotions, and improve overall well-being. Following the 2 webinars, participants receive 2 SMS text messages weekly for 5 weeks. Each SMS text message contains a brief review text, a reminder of the 5ways activities, and a list of example exercises for practicing the activities. Moreover, they provide a time schedule, allowing participants to select a suitable time for practicing the activities (eg, “On my way to work,” “during the weekends when I’m at home”).

**Table 1:** Intervention schedule.

Measurement schedule			Intervention activity
**Baseline assessment**			
	0-5 weeks before the intervention	Email with the questionnaire and a link to the main webinar	
	1	Main webinar (2 hours)	
	3	Email with a link to the booster webinar	
	4	Booster webinar (1 hour)	
**4 weeks’ assessment**			
	5	Email with the questionnaire	
	5	SMS text messages 1-2: Take notice + exercise	
	6	SMS text messages 3-4: Be active + exercises	
	7	SMS text messages 5-6: Keep learning + exercises	
	8	SMS text messages 7-8: Connect + exercises	
	9	SMS text messages 9-10: Give + exercises	
**Posttest assessment**			
	10-12	Email with a link to the questionnaire	

### Procedure

The webinars were delivered as live lectures and facilitated by a medical doctor. Participants who were unable to attend received a recorded version of the webinars. Web-based questionnaires were distributed by the project manager, and participants were assessed at 3 time points: (1) baseline measurement at the time of enrollment, (2) measurement after the booster webinar 4 weeks later, and (3) measurement after completing the full 10-week intervention. The control groups were assessed at the same time points as the intervention group. While most participants completed the baseline assessment at least 5 weeks before the intervention and within 2 weeks of the end of the intervention (posttest), a small subset experienced delays or misunderstandings, resulting in completion outside this time frame. Nonetheless, they were still included in the study. On average, it took participants 10 minutes to complete the questionnaires.

### Outcome Measures

To capture key aspects of well-being and mental health problems, we used various measures. Life satisfaction was assessed using the Satisfaction With Life Scale (SWLS), a well-established 5-item scale designed to gauge overall satisfaction with life [83]. The SWLS includes statements such as “I am satisfied with my life,” with responses provided on a 7-point scale ranging from 1 (strongly disagree) to 7 (strongly agree). In our sample, Cronbach α for the SWLS was .90, indicating high internal consistency. Besides, we used the 8-item Flourishing Scale (FS), which measures eudaimonic aspects of well-being [84]. This scale has excellent psychometric properties and includes statements such as “I lead a purposeful and meaningful life,” scored on the same 7-point scale as the SWLS. The estimated Cronbach α for the FS was .87, demonstrating strong internal reliability. In addition to the SWLS and FS, we used a set of questions from the National Quality of Life Survey to assess basic positive emotions (ie, happiness, engagement, calmness, and curiosity/interest) experienced over the past week. Participants rated each emotion on an 11-point scale ranging from 0 (not at all) to 10 (very much) [85]. The Cronbach α for the 4 positive emotions in our sample was .79, indicating good internal consistency. Notably, this set of emotional items is included in several large-scale national and regional surveys in Norway from 2019, with a total sample size exceeding 400,000. In addition to the individual scales, we aggregated the SWLS, FS, and positive emotion measures into a global well-being measure, which encompasses 3 crucial factors (cognitive, eudaimonic, and positive affect) in subjective well-being [86]. Furthermore, mental health was assessed using the Hopkins Symptom Checklist-8 (HSCL-8), an 8-item scale commonly used to gauge anxiety and depressive symptoms. The HSCL-8 includes questions such as whether participants feel “nervousness and shakiness inside” or “down or blue”. We used a 5-point response scale ranging from 1 (not being bothered at all) to 5 (bothering me a lot) [87]. To ensure consistency across measures, 1 item concerning hopelessness about the future was excluded from the HSCL-8 scale, as it was partially covered by other measures. The Cronbach α for the resulting 7-item HSCL (HSCL-7) was calculated to be .90, indicating high internal consistency. Before analysis, each of the 5 outcome measures (SWLS, FS, positive emotion, global well-being, and HSCL-8) was standardized with respect to the first measurement point. Specifically, we subtracted the mean at baseline and divided by the SD at baseline.

### Analytic Strategy

We used linear mixed (multilevel) models to investigate changes during the intervention period, using data from all assessments. Mixed modeling offers flexibility in handling missingness resulting from nonparticipation and dropout, as well as accounting for clustering caused by observations nested within individuals [88]. The time variable was coded as 0.0, 0.5, and 1.0 for baseline, booster, and posttest, respectively. The intervention group exhibited a greater increase in well-being and a decrease in mental health problems over time, as evidenced by a significant group × time interaction in the mixed models (α<.05). This interaction was interpreted as evidence for an intervention effect. Effect sizes were reported using Cohen *d* for all measures. In the primary analysis, we used an intention-to-treat approach, which included all participants who had completed more than 1 questionnaire within the intervention period. To assess the robustness of the results, we conducted additional analyses using only complete cases, which included participants who responded at all 3 time points (Multimedia Appendix 3). Furthermore, to explore the association between baseline levels and subsequent development, we fitted a model with a 3-way interaction between baseline level, time, and group (Multimedia Appendix 4).

## Results

### Characteristics of Participants

Table 2 presents the self-reported characteristics of the study participants at baseline. The majority of participants identified themselves as women (287/450, 63.8%), with a smaller proportion identifying as men (3 participants identified themselves as nonbinary). In addition, 393/450 (87.3%) participants reported having higher education (>high school). Chi-square analyses indicated no significant differences in age (*P*=.30), gender (*P*=.19), or education levels (*P*=.28) between the intervention and control groups. Across all groups, there was a moderate tendency for participants who dropped out at the posttest assessment to have lower SWLS scores at baseline.

**Table 2:** Participants characteristics (N=963).

Characteristics		Intervention group (n=450), n (%)	Control group (n=513), n (%)
**Age (years)**			
	18-39	161 (35.8)	175 (34.1)
	40-59	246 (54.7)	281 (54.8)
	≥60	43 (9.6)	57 (11.1)
**Gender**			
	Female	287 (63.8)	328 (63.9)
	Male	160 (35.6)	185 (36.1)
	Nonbinary	3 (0.7)	0 (0)
**Education**			
	Primary school	6 (1.3)	6 (1.2)
	High school	51 (11.3)	54 (10.5)
	≤4 years of college or university	93 (20.7)	131 (25.5)
	>4 years of college or university	300 (66.7)	322 (62.7)

### Outcomes

**Figure 2:**
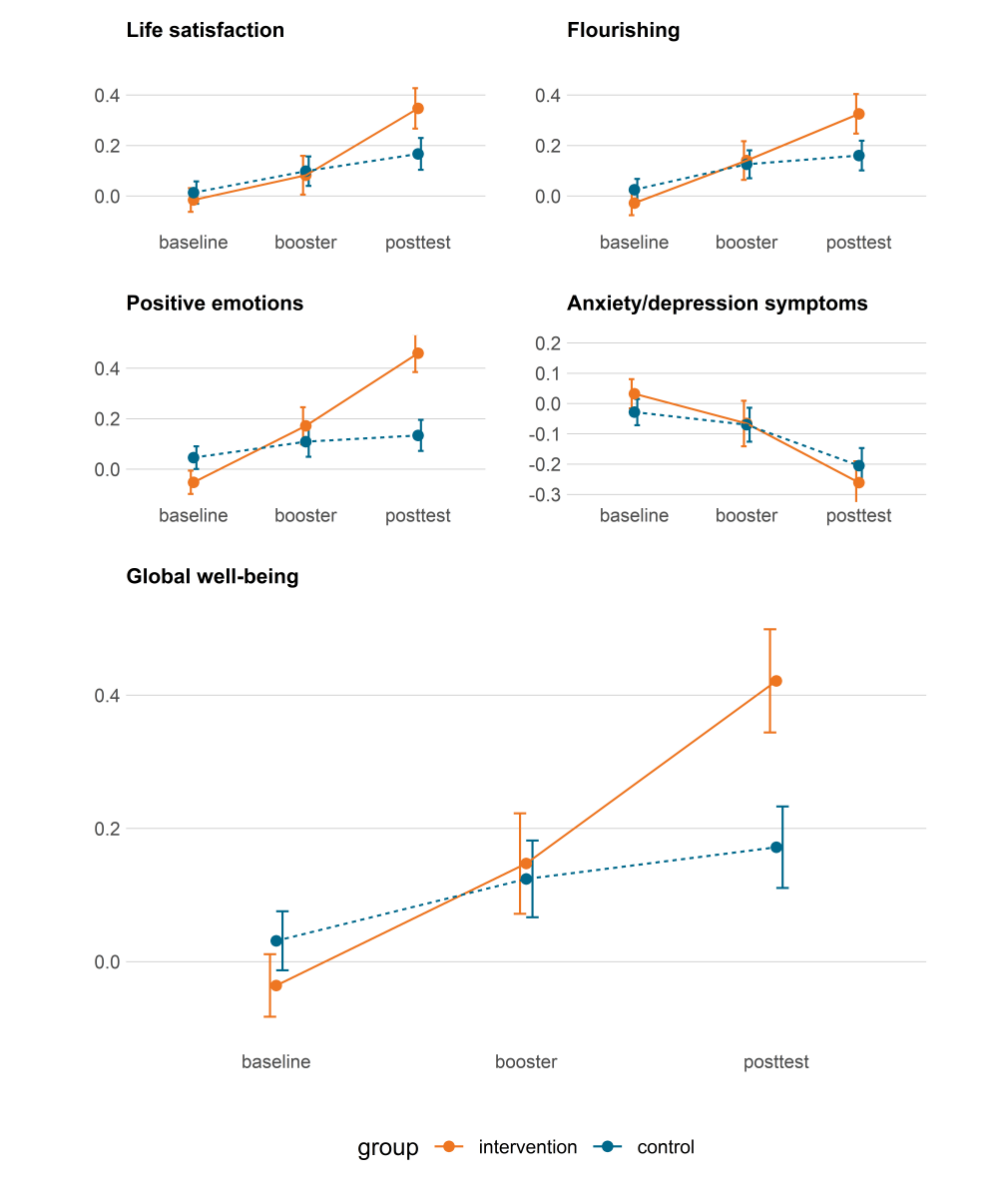
Mean scores for the intervention group and the control group for life satisfaction, flourishing, positive emotions, the 3 well-being outcomes combined (global well-being), and depression and anxiety symptoms at baseline, 4 weeks into the intervention (booster), and at posttest 10 weeks after baseline. The bars indicate SEs.

While there was a small trend toward more positive development in the active control group compared with the passive control group, these differences were not statistically significant for any of the outcome variables (SWLS, *P*=.97; flourishing, *P*=.44; positive emotions, *P*=.76; global well-being, *P*=.70; and HSCL, *P*=.15). Therefore, in the main analysis, the 2 control conditions were combined to increase statistical power and simplify interpretation. As reported in Table 3, multilevel analysis revealed a significantly greater increase from baseline to posttest in the intervention group compared with the control group for various outcome measures. Specifically, for the SWLS, the increase was significant (b=0.13, 95% CI 0.03-0.23; *P*=.001). Similarly, for the FS, positive emotions, and global well-being, the increases were also significant (FS: b=0.19, 95% CI 0.08-0.30; *P*=.001; positive emotions: b=0.43, 95% CI 0.27-0.60; *P*<.001; and global well-being: b=0.28, 95% CI 0.16-0.39; *P*<.001). The effect sizes for the intervention effect, calculated as Cohen *d*, were as follows: 0.30 (95% CI 0.11-0.49) for the SWLS, 0.32 (95% CI 0.12-0.51) for the FS, 0.49 (95% CI 0.29-0.68) for positive emotions, and 0.48 (95% CI 0.29-0.68) for global well-being. All of these effect sizes were statistically significant at *P*<.05. Furthermore, the multilevel analysis for HSCL-7 revealed a greater decrease from baseline to posttest in the intervention group compared with the control group (b=–0.17, 95% CI –0.30 to –0.04). This finding underscores the effectiveness of the intervention in reducing mental health problems. The significance level for the decrease in the HSCL-7 score from baseline to posttest was *P*=.001, with a Cohen *d* of –0.20 (95% CI –0.40 to –0.01). The results for all the outcomes are graphically presented in Figure 2. The attrition rate from baseline to posttest was 53.2% (516/969). Across all groups, there was a moderate tendency for participants who dropped out at the posttest assessment to have lower SWLS scores at baseline. To further investigate the robustness of the data, we reran the analyses with only the participants who had completed all 3 measurements (baseline, 4 weeks, and posttest). The results from the complete case analysis remained significant for all outcome measures, indicating the robustness of the findings (see Tables S6-S10 in Multimedia Appendix 3 for more details). Although we found no significant 3-way interaction between baseline levels, time, and group for any of the well-being outcomes, a significant interaction was observed for symptoms of depression and anxiety. This suggests that individuals with higher symptoms at baseline experienced a greater decrease in symptoms following the intervention compared with those with low symptom levels (see Multimedia Appendix 4 for more details).

**Table 3:** Results from the multilevel regression model of the 5 outcome measures with the effect size Cohen d for the intervention effect (*P*<.05).

Outcome measure		Intervention group			Control group			Intervention effect	
		n	Mean (SD)	n		Mean (SD)	Estimates (95% CI)^a^		Cohen *d* (95% CI)^b^
**Life satisfaction**							0.13 (0.03 to 0.23)		0.30 (0.11 to 0.50)
	Baseline	450	4.23 (1.28)	513		4.27 (1.27)			
	Booster	196	4.36 (1.37)	310		4.38 (1.31)			
	Posttest	171	4.69 (1.34)	282		4.46 (1.35)			
**Flourishing**							0.19 (0.08 to 0.30)		0.32 (0.12 to 0.51)
	Baseline	450	5.38 (1.16)	513		5.44 (1.12)			
	Booster	196	5.58 (1.22)	310		5.56 (1.11)			
	Posttest	171	5.79 (1.17)	282		5.6 (1.13)			
**Positive emotions**							0.43 (0.27 to 0.60)		0.49 (0.29 to 0.68)
	Baseline	450	26.16 (6.71)	513		26.83 (6.97)			
	Booster	196	27.69 (7.07)	310		27.26 (7.18)			
	Posttest	171	29.67 (6.7)	282		27.43 (7.10)			
**Global well-being**							0.28 (0.16 to 0.39)		0.48 (0.29 to 0.68)
	Baseline	450	–0.04 (1)	513		0.03 (1)			
	Booster	196	0.15 (1.06)	310		0.12 (1.01)			
	Posttest	171	0.42 (1.01)	282		0.17 (1.03)			
**Anxiety/depression symptoms**							–0.17 (–0.30 to –0.04)		–0.20 (–0.40 to –0.01)
	Baseline	450	2.22 (0.89)	513		2.17 (0.85)			
	Booster	196	2.14 (0.92)	310		2.13 (0.86)			
	Posttest	171	1.97 (0.80)	282		2.02 (0.85)			

^a^Estimates for the intervention effect from baseline to posttest

^b^Cohen *d* for the intervention effect from baseline to posttest.

### Adherence

To assess adherence, participants were asked whether they watched the webinars in their entirety or partially, either live or recorded. Moreover, they were asked whether they engaged in the exercises sent to them via SMS text messages. Of the 171 participants in the intervention group, adherence was assessed in 134 participants, while 37 participants were not asked about this because of a delay in incorporating the relevant questions in the questionnaires. As we could not identify attendees or register them through the webinar platform, nor could we directly measure whether they received the SMS text messages, self-reporting became the sole method for assessing adherence. This realization emerged after the initial 37 participants had provided their responses, which is why they were excluded from this assessment. At the posttest, 117/134 (87.3%) of the participants reported having watched the entire main webinar, or parts of it, either live or recorded. In addition, 105/134 (78.3%) reported having watched the entire or parts of the booster webinar, either live or recorded. Furthermore, 126/134 (94.0%) reported engaging in some or all of the exercises sent to them in the weekly SMS text messages.

## Discussion

### Principal Findings

The findings from this study suggest that 5waysA is an effective web-based intervention for promoting well-being and mental health. This aligns with previous research indicating the effectiveness of internet-based interventions [16,19,24]. While the effect sizes may be small classified according to Cohen’s *d*, they are still noteworthy and comparable to psychological intervention studies that detect small to moderate effects [35,38]. Moreover, the effect sizes observed in this study are comparable to those reported for psychotherapy or antidepressant medication in treating depression [89-91]. Indeed, meta-analyses have highlighted the importance of control conditions in intervention studies [81,82]. However, in this study, the distinction between passive and active control conditions did not significantly impact the observed effects of the 5waysA intervention. This underscores the effectiveness of the intervention itself rather than the specific type of control condition used. These positive outcomes align with existing research demonstrating that psychological interventions, regardless of their theoretical framework or delivery method, can effectively improve well-being and reduce symptoms of anxiety and depression [35-38]. This study provides additional support for the effectiveness of the Five Ways to Wellbeing framework in enhancing well-being and mental health among the general population. Previous examinations of the framework have primarily occurred in nonintervention settings [74] or with hospitalized patients [75], rather than as a web-based intervention aimed at the broader population.

The significance of our study lies in its ability to demonstrate effects across all core dimensions of subjective well-being—cognitive evaluations, emotional experiences, and eudaimonic aspects. This breadth of impact suggests relevance for individuals facing various struggles or experiencing a state of languishing within the multidimensional construct of well-being. Moreover, our findings indicate that participants with a higher number of symptoms associated with depression and anxiety were more responsive to the intervention than those with few symptoms, suggesting that the 5waysA intervention may also be beneficial for clinical or subclinical populations. The alignment between the 5 action domains in the Five Ways to Wellbeing framework and the basic psychological needs in self-determination theory, such as autonomy, relatedness, and competence, as well as the core dimensions of the training-based framework by Dahl et al [32], including awareness, connection, insight, and purpose, is noteworthy. These perspectives share the common goal of guiding individuals toward actions that yield observable and meaningful positive consequences for their overall well-being, health, and capacity to thrive and flourish. The potential to guide individuals toward actions and promote cognitive and behavioral coping strategies is often considered a common mechanism of change across psychological interventions. In web-based interventions, this can be facilitated through reminders sent via emails, apps, or SMS text messages, promoting user self-reliance in a cost-effective and easily implemented manner. The strong emphasis on self-management and self-empowerment may be one of several mechanisms that contribute to making internet-based interventions comparable to on-site treatments and interventions. However, the active mechanisms of change in web-based interventions are still not fully understood.

Web-based interventions show promise in their ability to reach a broad audience. A recent review and meta-analysis [15] suggested that internet-based interventions for treating mental health problems are likely cost-effective, although the heterogeneity of studies makes generalizability challenging. In addition, prevention is known to be more cost-effective than treatment. The paradox of prevention [13] suggests that universal, broad health and well-being–promoting interventions aimed at the general population can be highly beneficial. Even modest improvements in many individuals can have a powerful impact at the population level. The 5waysA intervention, with its web-based delivery mode, has the potential to reach many people efficiently. It can be implemented through primary care, workplaces, municipal websites, high schools, universities, and other institutions.

Enhanced well-being can potentially impact both mental and physical health. The 5waysA intervention, as a transdiagnostic, multidimensional intervention, may represent a cost-effective measure capable of promoting overall well-being and mental health. Its low administration costs and web-based format make it suitable for an economically pressured health care service. This solution is also suitable for individuals who live in remote areas without easy access to health care services, are physically disabled, have busy schedules, or simply prefer web-based options (eg, young people). In addition, because this study was conducted during the COVID-19 outbreak, which had a significant impact on well-being, we anticipate that the intervention could also prove effective in future pandemics and lockdowns.

### Strengths and Limitations

This study possesses several strengths, including its relatively large sample size, utilization of various control conditions, incorporation of validated outcome measures, and the use of items/scales enabling comparison with national surveys conducted concurrently. However, it also exhibits some limitations. As anticipated with web-based interventions [92], the attrition rate was relatively high (516/969, 53.2%). In terms of demographic variables, there were no systematic differences observed regarding the participants lost between groups. However, between the baseline and booster webinar, there was a higher dropout rate observed in the intervention group compared with the control group. The reason behind the increased dropout rate in the intervention group during this period remains unclear to us. The higher dropout rate in the intervention group during this period may be attributed to elevated expectations, such as potential disappointment following the webinar attendance, or reluctance to engage with the intervention if the webinar was missed, while the control group remained expectant. Future studies should delve deeper into strategies for preventing attrition in web-based interventions, exploring the intricate interplay among technological challenges, levels of support, and reminders, alongside individual user demographics [93,94].

The control condition in this study comprises both an active and a passive group, thus demonstrating a high-quality design. However, future studies should contemplate comparing the 5waysA with other web-based interventions within the multicomponent positive psychology interventions spectrum (eg, Chilver and Gatt [42]) to mitigate uncertainties surrounding its true effectiveness.

In addition, an important limitation of this study pertains to the short follow-up time. Future research should also explore potential mediating mechanisms. The representativeness of the sample is suboptimal, with 393/450 (87.3%) participants reporting a higher level of education, whereas the population mean is 36.9% [95]. However, the participants’ mean baseline SWLS (sum) score of 21.25 was notably lower than the population mean of 25.21 [53], suggesting that our sample population might be at risk of transitioning toward compromised mental health or a state of languishing. Moreover, our results appear to indicate that they constitute a subset of the population that responds positively to well-being–promoting interventions. Their improved well-being may subsequently enhance their ability to manage work-life balance and caregiving responsibilities and shield them from detrimental stress. Indeed, future studies should evaluate the effectiveness of the 5waysA intervention in a more diverse sample, encompassing marginalized populations and specific illness groups. It might also be suitable to incorporate the Five Ways to Wellbeing concept into a population health approach, where interventions are less focused on individual behavior change and more integrated into local structures as societal measures and initiatives. This approach could ensure equal accessibility of the concept to various societal groups. Future research should assess the effectiveness of these types of Five Ways to Wellbeing measures.

### Conclusions

Our results indicate that the web-based 5waysA intervention is an effective and promising intervention for promoting well-being among the general population. This study offers policy makers and local stakeholders a scalable, potentially cost-effective, and efficacious health promotion intervention with the potential for widespread impact. The 5waysA intervention targets the individual level, but its benefits can have meaning for individuals, groups, and societies as well.

## Appendix

Multimedia Appendix 1 CONSORT-EHEALTH checklist (V 1.6.1).

Multimedia Appendix 2 Information letter, written consent , and questionnaires used in the study (in Norwegian).

Multimedia Appendix 3 Results of the mixed models 1-5.

Multimedia Appendix 4 Results of the mixed models 1-6.

## Abbreviations

5waysA: Five Ways to Wellbeing for All
CONSORT: Consolidated Standards of Reporting Trials
FS: Flourishing Scale
GDPR: General Data Protection Regulation
HSCL-7: 7-item Hopkins Symptom Checklist
HSCL-8: 8-item Hopkins Symptom Checklist
REK: The Regional Committees for Health Research Ethics
SWLS: Satisfaction With Life Scale
TSD: Services for Sensitive Data
USIT: University Information Technology Center
WHO: World Health Organization

## Data Availability

In accordance with ethical approval from The Regional Committees for Health Research Ethics (REK), we are currently not permitted to share data.
