# Supplementary material for: A Web-Based Intervention Using "Five Ways to Wellbeing" to Promote Well-Being and Mental Health: Randomized Controlled Trial
Source: JMIR Ment Health. 2024 May 20;11:e49050. doi: 10.2196/49050 (PMC11148523; doi:10.2196/49050)
Supplement: Multimedia Appendix 2 [file mental_v11i1e49050_app2.docx]

**Appendix 2**

### This is a Multimedia Appendix to a full manuscript to be published in the J Med Internet Res Mental Health with the title “The Web-based Intervention “Five Ways to Wellbeing” Promotes Well-being and Mental Health: A Randomized Controlled Trial”

Information letter and written consent (in Norwegian):

<https://nettskjema.no/user/form/185925>

Questionnaires used (in Norwegian):

<https://nettskjema.no/user/form/314349/view>
