# Supplementary material for: A Web-Based Intervention Using "Five Ways to Wellbeing" to Promote Well-Being and Mental Health: Randomized Controlled Trial
Source: JMIR Ment Health. 2024 May 20;11:e49050. doi: 10.2196/49050 (PMC11148523; doi:10.2196/49050)
Supplement: Multimedia Appendix 3 [file mental_v11i1e49050_app3.docx]

**Appendix 3**

### This is a Multimedia Appendix to a full manuscript to be published in the J Med Internet Res Mental Health with the title “The Web-based Intervention “Five Ways to Wellbeing” Promotes Well-being and Mental Health: A Randomized Controlled Trial”

.

|  | **Model 1** | **Model 2** | **Model 3** | **Model 4** | **Model 5** |
| --- | --- | --- | --- | --- | --- |
| *Predictors* | *Estimates* | *Estimates* | *Estimates* | *Estimates* | *Estimates* |
| (Intercept) | 0.10 ^**^ | 0.04 | 0.04 | 0.04 | 0.04 |
| Time |  | 0.16 ^***^ | 0.16 ^***^ | 0.10 ^**^ | 0.10 ^**^ |
| Time* Intervention |  |  |  | 0.13 ^*^ | 0.13 ^*^ |
| Intervention |  |  |  |  | -0.00 |
| **Random Effects** | | | | | |
| σ^2^ | 0.16 | 0.15 | 0.15 | 0.15 | 0.15 |
| τ_00_ | 0.91 | 0.91 | 0.87 | 0.87 | 0.87 |
| τ_11_ |  |  | 0.03 | 0.02 | 0.02 |
| ρ_01_ |  |  | 0.30 | 0.35 | 0.35 |
| AIC | 3292.440 | 3255.600 | 3251.966 | 3247.536 | 3249.536 |
| * *P*<0.05, ** *P*<0.01, *** *P*<0.001. | | | | | |

**Table 1A**: Mixed model results for the effects of time, intervention, and their interaction in SWLS. Model 1 includes a fixed and random intercept, Model 2 adds the fixed effect of time, Model 3 also incorporates a random slope for time, Model 4 adds a time by group interaction, and Model 5 we also control for group differences. The table presents estimates of fixed effects, random effects, and model fit (AIC). Significance levels: * *P*<0.05, ** *P*<0.01, *** *P*<0.001.

**Table 1B**: Mixed model results for the effects of time, intervention, and their interaction in the Flourishing scale as outcome measure. Model 1 includes a fixed and random intercept, Model 2 adds the fixed effect of time, Model 3 also incorporates a random slope for time, Model 4 adds a time by group interaction, and Model 5 we also control for group differences. The table presents estimates of fixed effects, random effects, and model fit (AIC). Significance levels: * *P*<0.05, ** *P*<0.01, *** *P*<0.001.

|  | **Model 1** | **Model 2** | **Model 3** | **Model 4** | **Model 5** |
| --- | --- | --- | --- | --- | --- |
| *Predictors* | *Estimates* | *Estimates* | *Estimates* | *Estimates* | *Estimates* |
| (Intercept) | 0.11 ^**^ | 0.04 | 0.04 | 0.04 | 0.05 |
| Time |  | 0.16 ^***^ | 0.16 ^***^ | 0.09 ^**^ | 0.09 ^*^ |
| Time * Intervention |  |  |  | 0.18 ^**^ | 0.19 ^**^ |
| Intervention |  |  |  |  | -0.04 |
| **Random Effects** | | | | | |
| σ^2^ | 0.17 | 0.17 | 0.14 | 0.14 | 0.14 |
| τ_00_ | 0.84 | 0.84 | 0.86 | 0.86 | 0.86 |
| τ_11_ |  |  | 0.11 | 0.10 | 0.10 |
| ρ_01_ |  |  | -0.12 | -0.12 | -0.12 |
| AIC | 3331.343 | 3296.949 | 3289.707 | 3281.012 | 3282.786 |
| * *P*<0.05, ** *P*<0.01, *** *P*<0.001. | | | | | |

|  | **Model 1** | **Model 2** | **Model 3** | **Model 4** | **Model 5** |
| --- | --- | --- | --- | --- | --- |
| *Predictors* | *Estimates* | *Estimates* | *Estimates* | *Estimates* | *Estimates* |
| (Intercept) | 0.12 ^**^ | 0.03 | 0.03 | 0.03 | 0.08 |
| Time |  | 0.21 ^***^ | 0.20 ^***^ | 0.06 | 0.04 |
| Time * Intervention |  |  |  | 0.38 ^***^ | 0.43 ^***^ |
| Intervention |  |  |  |  | -0.13 |
| **Random Effects** | | | | | |
| σ^2^ | 0.40 | 0.39 | 0.35 | 0.35 | 0.35 |
| τ_00_ | 0.65 | 0.66 | 0.68 | 0.68 | 0.68 |
| τ_11_ |  |  | 0.16 _._ | 0.12 _._ | 0.12 _._ |
| ρ_01_ |  |  | -0.15 | -0.14 | -0.13 |
| AIC | 4080.641 | 4056.366 | 4055.158 | 4033.225 | 4032.619 |
| * *P*<0.05, ** *P*<0.01, *** *P*<0.001. | | | | | |

**Table 1C**: Mixed model results for the effects of time, intervention, and their interaction in Positive emotions as outcome measure. Model 1 includes a fixed and random intercept, Model 2 adds the fixed effect of time, Model 3 also incorporates a random slope for time, Model 4 adds a time by group interaction, and Model 5 we also control for group differences. The table presents estimates of fixed effects, random effects, and model fit (AIC). Significance levels: * *P*<0.05, ** *P*<0.01, *** *P*<0.001.

**Table 1D**: Mixed model results for the effects of time, intervention, and their interaction in the Global wellbeing as outcome measure. Model 1 includes a fixed and random intercept, Model 2 adds the fixed effect of time, Model 3 also incorporates a random slope for time, Model 4 adds a time by group interaction, and Model 5 we also control for group differences. The table presents estimates of fixed effects, random effects, and model fit (AIC). Significance levels: * *P*<0.05, ** *P*<0.01, *** *P*<0.001.

|  | **Model 1** | **Model 2** | **Model 3** | **Model 4** | **Model 5** |
| --- | --- | --- | --- | --- | --- |
| *Predictors* | *Estimates* | *Estimates* | *Estimates* | *Estimates* | *Estimates* |
| (Intercept) | 0.12 ^**^ | 0.04 | 0.04 | 0.04 | 0.07 |
| Time |  | 0.19 ^***^ | 0.19 ^***^ | 0.09 ^**^ | 0.09 ^*^ |
| Time * Intervention |  |  |  | 0.26 ^***^ | 0.28 ^***^ |
| Intervention |  |  |  |  | -0.07 |
|  |  |  |  |  |  |
| **Random Effects** | | | | | |
| σ^2^ | 0.18 | 0.17 | 0.14 | 0.15 | 0.15 |
| τ_00_ | 0.86 | 0.86 | 0.87 | 0.87 | 0.87 |
| τ_11_ |  |  | 0.12 | 0.10 | 0.10 |
| ρ_01_ |  |  | -0.06 | -0.05 | -0.05 |
| AIC | 3403.450 | 3354.536 | 3344.707 | 3324.641 | 3325.919 |
| * *P*<0.05, ** *P*<0.01, *** *P*<0.001. | | | | | |

**Table 1E**: Mixed model results for the effects of time, intervention, and their interaction in the SCL as outcome measure. Model 1 includes a fixed and random intercept, Model 2 adds the fixed effect of time, Model 3 also incorporates a random slope for time, Model 4 adds a time by group interaction, and Model 5 we also control for group differences. The table presents estimates of fixed effects, random effects, and model fit (AIC). Significance levels: * *P*<0.05, ** *P*<0.01, *** *P*<0.001.

|  | **Model 1** | | **Model 2** | | **Model 3** | | **Model 4** | | **Model 5** | |
| --- | --- | --- | --- | --- | --- | --- | --- | --- | --- | --- |
| *Predictors* | *Estimates* |  | *Estimates* |  | *Estimates* |  | *Estimates* |  | *Estimates* |  |
| (Intercept) | -0.08 ^*^ |  | -0.01 |  | -0.01 |  | -0.01 |  | -0.05 |  |
| Time |  |  | -0.17 ^***^ |  | -0.17 ^***^ |  | -0.12 ^**^ |  | -0.11 ^*^ |  |
| Time * Intervention |  |  |  |  |  |  | -0.14 ^*^ |  | -0.17 ^**^ |  |
| Intervention |  |  |  |  |  |  |  |  | 0.09 |  |
| **Random Effects** | | | | | | | | | | |
| σ^2^ | 0.25 | | 0.25 | | 0.23 | | 0.23 | | 0.23 | |
| τ_00_ | 0.72 | | 0.72 | | 0.79 | | 0.79 | | 0.79 | |
| τ_11_ |  | |  | | 0.08 | | 0.07 | | 0.07 | |
| ρ_01_ |  | |  | | -0.35 | | -0.34 | | -0.34 | |
| AIC | 3645.871 | | 3623.911 | | 3622.473 | | 3622.862 | | 3626.741 | |
| ** p<0.05   ** p<0.01   *** p<0.001* | | | | | | | | | | |

COMPLETE CASES

**Table 1F**: Mixed model results for the effects of time, intervention, and their interaction in SWLS for complete cases. Model 1 includes a fixed and random intercept, Model 2 adds the fixed effect of time, Model 3 also incorporates a random slope for time, Model 4 adds a time by group interaction, and Model 5 we also control for group differences. The table presents estimates of fixed effects, random effects, and model fit (AIC). Significance levels: * * *P*<0.05, ** *P*<0.01, *** *P*<0.001.

|  | **Model 1** | **Model 2** | **Model 3** | **Model 4** | **Model 5** |
| --- | --- | --- | --- | --- | --- |
| *Predictors* | *Estimates* | *Estimates* | *Estimates* | *Estimates* | *Estimates* |
| (Intercept) | 0.12 ^*^ | 0.04 | 0.04 | 0.04 | -0.00 |
| Time |  | 0.16 ^***^ | 0.16 ^***^ | 0.27 ^***^ | 0.27 ^***^ |
| Time * Intervention |  |  |  | -0.17 ^**^ | -0.18 ^**^ |
| Intervention |  |  |  |  | 0.07 |
| **Random Effects** | | | | | |
| σ^2^ | 0.16 | 0.16 | 0.15 | 0.15 | 0.15 |
| τ_00_ | 0.91 | 0.91 | 0.88 | 0.88 | 0.88 |
| τ_11_ |  |  | 0.04 | 0.04 | 0.04 |
| ρ_01_ |  |  | 0.14 | 0.16 | 0.16 |
| AIC | 2213.817 | 2185.399 | 2185.020 | 2179.556 | 2181.111 |
| * *P*<0.05, ** *P*<0.01, *** *P*<0.001. | | | | | |

|  | **Model 1** | **Model 2** | **Model 3** | **Model 4** | **Model 5** |
| --- | --- | --- | --- | --- | --- |
| *Predictors* | *Estimates* | *Estimates* | *Estimates* | *Estimates* | *Estimates* |
| (Intercept) | 0.14 ^**^ | 0.07 | 0.07 | 0.07 | -0.02 |
| Time |  | 0.15 ^***^ | 0.15 ^***^ | 0.28 ^***^ | 0.29 ^***^ |
| Time * Intervention |  |  |  | -0.19 ^**^ | -0.22 ^**^ |
| Intervention |  |  |  |  | 0.14 |
| **Random Effects** | | | | | |
| σ^2^ | 0.17 | 0.16 | 0.14 | 0.14 | 0.14 |
| τ_00_ | 0.86 | 0.86 | 0.88 | 0.88 | 0.88 |
| τ_11_ |  |  | 0.11 | 0.10 | 0.10 |
| ρ_01_ |  |  | -0.12 | -0.11 | -0.11 |
| AIC | 2228.684 | 2204.611 | 2198.384 | 2191.504 | 2191.652 |
| * *P*<0.05, ** *P*<0.01, *** *P*<0.001. | | | | | |

**Table 1G**: Mixed model results for the effects of time, intervention, and their interaction in Flourishing scale for complete cases. Model 1 includes a fixed and random intercept, Model 2 adds the fixed effect of time, Model 3 also incorporates a random slope for time, Model 4 adds a time by group interaction, and Model 5 we also control for group differences. The table presents estimates of fixed effects, random effects, and model fit (AIC). Significance levels: * *P*<0.05, ** *P*<0.01, *** *P*<0.001.

**Table 1H**: Mixed model results for the effects of time, intervention, and their interaction in Positive Emotions for complete cases. Model 1 includes a fixed and random intercept, Model 2 adds the fixed effect of time, Model 3 also incorporates a random slope for time, Model 4 adds a time by group interaction, and Model 5 we also control for group differences. The table presents estimates of fixed effects, random effects, and model fit (AIC). Significance levels: * *P*<0.05, ** *P*<0.01, *** *P*<0.001.

|  | **Model 1** | **Model 2** | **Model 3** | **Model 4** | **Model 5** |
| --- | --- | --- | --- | --- | --- |
| *Predictors* | *Estimates* | *Estimates* | *Estimates* | *Estimates* | *Estimates* |
| (Intercept) | 0.14 ^**^ | 0.05 | 0.05 | 0.05 | -0.06 |
| Time |  | 0.19 ^***^ | 0.19 ^***^ | 0.41 ^***^ | 0.45 ^***^ |
| Time * Intervention |  |  |  | -0.34 ^***^ | -0.41 ^***^ |
| Intervention |  |  |  |  | 0.16 |
| **Random Effects** | | | | | |
| σ^2^ | 0.41 | 0.40 | 0.35 | 0.35 | 0.35 |
| τ_00_ | 0.67 | 0.67 | 0.72 | 0.72 | 0.72 |
| τ_11_ |  |  | 0.20 | 0.16 | 0.16 |
| ρ_01_ |  |  | -0.22 | -0.21 | -0.20 |
| AIC | 2838.293 | 2823.699 | 2821.834 | 2809.900 | 2809.665 |
| * *P*<0.05, ** *P*<0.01, *** *P*<0.001. | | | | | |

**Table 1I**: Mixed model results for the effects of time, intervention, and their interaction in Global Wellbeing for complete cases. Model 1 includes a fixed and random intercept, Model 2 adds the fixed effect of time, Model 3 also incorporates a random slope for time, Model 4 adds a time by group interaction, and Model 5 we also control for group differences. The table presents estimates of fixed effects, random effects, and model fit (AIC). Significance levels: * *P*<0.05, ** *P*<0.01, *** *P*<0.001.

|  | **Model 1** | **Model 2** | **Model 3** | **Model 4** | **Model 5** |
| --- | --- | --- | --- | --- | --- |
| *Predictors* | *Estimates* | *Estimates* | *Estimates* | *Estimates* | *Estimates* |
| (Intercept) | 0.15 ^**^ | 0.06 | 0.06 | 0.06 | -0.04 |
| Time |  | 0.19 ^***^ | 0.19 ^***^ | 0.36 ^***^ | 0.38 ^***^ |
| Time * Intervention |  |  |  | -0.27 ^***^ | -0.30 ^***^ |
| Intervention |  |  |  |  | 0.15 |
| **Random Effects** | | | | | |
| σ^2^ | 0.19 | 0.18 | 0.14 | 0.14 | 0.14 |
| τ_00_ | 0.87 | 0.87 | 0.89 | 0.89 | 0.89 |
| τ_11_ |  |  | 0.13 | 0.11 | 0.11 |
| ρ_01_ |  |  | -0.12 | -0.11 | -0.11 |
| AIC | 2300.384 | 2266.150 | 2257.355 | 2242.861 | 2242.892 |
| * *P*<0.05, ** *P*<0.01, *** *P*<0.001. | | | | | |

**Table 1J**: Mixed model results for the effects of time, intervention, and their interaction in the SCL for complete cases. Model 1 includes a fixed and random intercept, Model 2 adds the fixed effect of time, Model 3 also incorporates a random slope for time, Model 4 adds a time by group interaction, and Model 5 we also control for group differences. The table presents estimates of fixed effects, random effects, and model fit (AIC). Significance levels: * *P*<0.05, ** *P*<0.01, *** *P*<0.001.

|  | **Model 1** | **Model 2** | **Model 3** | **Model 4** | **Model 5** |
| --- | --- | --- | --- | --- | --- |
| *Predictors* | *Estimates* | *Estimates* | *Estimates* | *Estimates* | *Estimates* |
| (Intercept) | -0.12 ^*^ | -0.02 | -0.02 | -0.02 | 0.11 |
| Time |  | -0.20 ^***^ | -0.20 ^***^ | -0.16 ^***^ | 0.08 ^*^ |
| Time * Intervention |  |  |  | -0.11 | 0.30 ^***^ |
| Intervention |  |  |  |  | -0.15 |
| **Random Effects** | | | | | |
| σ^2^ | 0.26 | 0.25 | 0.24 | 0.24 | 0.14 |
| τ_00_ | 0.78 | 0.78 | 0.86 | 0.86 | 0.89 |
| τ_11_ |  |  | 0.08 | 0.07 | 0.11 |
| ρ_01_ |  |  | -0.36 | -0.36 | -0.11 |
| AIC | 2542.350 | 2520.842 | 2520.327 | 2523.369 | 2258.327 |
| ** p<0.05   ** p<0.01   *** p<0.001* | | | | | |
