# Supplementary material for: A Web-Based Intervention Using "Five Ways to Wellbeing" to Promote Well-Being and Mental Health: Randomized Controlled Trial
Source: JMIR Ment Health. 2024 May 20;11:e49050. doi: 10.2196/49050 (PMC11148523; doi:10.2196/49050)
Supplement: Multimedia Appendix 4 [file mental_v11i1e49050_app4.docx]

### Appendix 4

### This is a Multimedia Appendix to a full manuscript to be published in the J Med Internet Res Mental Health with the title “The Web-based Intervention “Five Ways to Wellbeing” Promotes Well-being and Mental Health: A Randomized Controlled Trial”

|  | **Model 1** | **Model 2** | **Model 3** | **Model 4** | **Model 5** | **Model 6** |
| --- | --- | --- | --- | --- | --- | --- |
| *Predictors* | *Estimates* | *Estimates* | *Estimates* | *Estimates* | *Estimates* | *Estimates* |
| (Intercept) | 0.10 ^**^ | 0.04 | 0.04 | 0.04 | 0.04 | 0.01 |
| Time |  | 0.16 ^***^ | 0.16 ^***^ | 0.10 ^**^ | 0.10 ^**^ | 0.12 ^***^ |
| Time*Intervention |  |  |  | 0.13 ^*^ | 0.13 ^*^ | 0.10 |
| Intervention |  |  |  |  | -0.00 | -0.01 |
| Baseline SWLS |  |  |  |  |  | 0.97 ^***^ |
| Time * Baseline SWLS |  |  |  |  |  | -0.11 ^**^ |
| Intervention * Baseline SWLS |  |  |  |  |  | 0.02 |
| Time* Intervention * Baseline SWLS |  |  |  |  |  | 0.00 |
| **Random Effects** | | | | | | |
| σ^2^ | 0.16 | 0.15 | 0.15 | 0.15 | 0.15 | 0.07 |
| τ_00_ | 0.91 | 0.91 | 0.87 | 0.87 | 0.87 | 0.00 |
| τ_11_ |  |  | 0.03 | 0.02 | 0.02 | 0.30 |
| ρ_01_ |  |  | 0.30 | 0.35 | 0.35 | 1.00 |
| AIC | 3297.063 | 3265.768 | 3262.094 | 3261.775 | 3267.008 | 1291.951 |
| ** p<0.05   ** p<0.01   *** p<0.001* | | | | | | |

**Table 2A**: Mixed model results for the effects of time, intervention, and their interaction in SWLS. Model 1 includes a fixed and random intercept, Model 2 adds the fixed effect of time, Model 3 also incorporates a random slope for time, Model 4 adds a time by group interaction, Model 5 we also control for group differences and Model 6 fits a model with a three-way interaction between baseline level, time, and group. The table presents estimates of fixed effects, random effects, and model fit (AIC). Significance levels: * *P*<0.05, ** *P*<0.01, *** *P*<0.001.

|  | **Model 1** | | **Model 2** | | **Model 3** | | **Model 4** | | **Model 5** | | **Model 6** | | |
| --- | --- | --- | --- | --- | --- | --- | --- | --- | --- | --- | --- | --- | --- |
| *Predictors* | *Estimates* |  | *Estimates* |  | *Estimates* |  | *Estimates* |  | *Estimates* |  | *Estimates* | |  |
| (Intercept) | 0.11 ^**^ |  | 0.04 |  | 0.04 |  | 0.04 |  | 0.05 |  | | 0.01 |  |
| Time |  |  | 0.16 ^***^ |  | 0.16 ^***^ |  | 0.09 ^**^ |  | 0.09 ^*^ |  | | 0.11 ^**^ |  |
| Time * Intervensjon |  |  |  |  |  |  | 0.18 ^**^ |  | 0.19 ^**^ |  | | 0.18 ^**^ |  |
| Intervention |  |  |  |  |  |  |  |  | -0.04 |  | | -0.01 |  |
| Baseline Flourishing |  |  |  |  |  |  |  |  |  |  | | 0.97 ^***^ |  |
| Time * Baseline Flourishing |  |  |  |  |  |  |  |  |  |  | | -0.17^***^ |  |
| Intervention * Baseline Flourishing |  |  |  |  |  |  |  |  |  |  | | 0.02 |  |
| Time * Intervention * Baseline Flourishing |  |  |  |  |  |  |  |  |  |  | | -0.07 |  |
| **Random Effects** | | | | | | | | | | | | | |
| σ^2^ | 0.17 | | 0.17 | | 0.14 | | 0.14 | | 0.14 | | 0.06 | | |
| τ_00_ | 0.84 | | 0.84 | | 0.86 | | 0.86 | | 0.86 | | 0.00 | | |
| τ_11_ |  | |  | | 0.11 | | 0.10 | | 0.10 | | 0.35 | | |
| ρ_01_ |  | |  | | -0.12 | | -0.12 | | -0.12 | | 1.00 | | |
| AIC | 3336.039 | | 3307.100 | | 3299.728 | | 3295.034 | | 3300.046 | | 1292.099 | | |
| ** p<0.05   ** p<0.01   *** p<0.001* | | | | | | | | | | | | | |

**Table 2B**: Mixed model results for the effects of time, intervention, and their interaction in Flourishing. Model 1 includes a fixed and random intercept, Model 2 adds the fixed effect of time, Model 3 also incorporates a random slope for time, Model 4 adds a time by group interaction, Model 5 we also control for group differences and Model 6 fits a model with a three-way interaction between baseline level, time, and group. The table presents estimates of fixed effects, random effects, and model fit (AIC). Significance levels: * *P*<0.05, ** *P*<0.01, *** *P*<0.001.

**Table 2C**: Mixed model results for the effects of time, intervention, and their interaction in Positive Emotions. Model 1 includes a fixed and random intercept, Model 2 adds the fixed effect of time, Model 3 also incorporates a random slope for time, Model 4 adds a time by group interaction, Model 5 we also control for group differences and Model 6 fits a model with a three-way interaction between baseline level, time, and group. The table presents estimates of fixed effects, random effects, and model fit (AIC). Significance levels: * *P*<0.05, ** *P*<0.01, *** *P*<0.001.

|  | **Model 1** | | **Model 2** | | **Model 3** | | **Model 4** | | **Model 5** | | **Model 6** | | | |  |
| --- | --- | --- | --- | --- | --- | --- | --- | --- | --- | --- | --- | --- | --- | --- | --- |
| *Predictors* | *Estimates* |  | *Estimates* |  | *Estimates* |  | *Estimates* |  | *Estimates* |  | *Estimates* | |  | |  |
| (Intercept) | 0.12 ^**^ |  | 0.03 |  | 0.03 |  | 0.03 |  | 0.08 |  | | 0.01 | |  | |
| Time |  |  | 0.21 ^***^ |  | 0.20 ^***^ |  | 0.06 |  | 0.04 |  | | 0.07 | |  | |
| Time* Intervention |  |  |  |  |  |  | 0.38 ^***^ |  | 0.43 ^***^ |  | | 0.37 ^***^ | |  | |
| Intervention |  |  |  |  |  |  |  |  | -0.13 |  | | -0.01 | |  | |
| Baseline positive emotions |  |  |  |  |  |  |  |  |  |  | | 0.97 ^***^ | |  | |
| Time * Baseline positive emotions |  |  |  |  |  |  |  |  |  |  | | -0.43 ^***^ | |  | |
| Intervention * Baseline positive emotions |  |  |  |  |  |  |  |  |  |  | | -0.02 | |  | |
| Time * Intervention * Baseline positive emotions |  |  |  |  |  |  |  |  |  |  | | -0.01 | |  | |
| **Random Effects** | | | | | | | | | | | | | | |  |
| σ^2^ | 0.40 | | 0.39 | | 0.35 | | 0.35 | | 0.35 | | 0.16 | | | |  |
| τ_00_ | 0.65 | | 0.66 | | 0.68 | | 0.68 | | 0.68 | | 0.00 | | | |  |
| τ_11_ |  | |  | | 0.16 | | 0.12 | | 0.12 | | 0.61 | | | |  |
| ρ_01_ |  | |  | | -0.15 | | -0.14 | | -0.13 | | 1.00 | | | |  |
| AIC | 4085.437 | | 4065.775 | | 4064.479 | | 4045.909 | | 4048.512 | | 2612.495 | | | |  |
| ** p<0.05   ** p<0.01   *** p<0.001* | | | | | | | | | | | | | | |  |

**Table 2D**: Mixed model results for the effects of time, intervention, and their interaction in Global wellbeing. Model 1 includes a fixed and random intercept, Model 2 adds the fixed effect of time, Model 3 also incorporates a random slope for time, Model 4 adds a time by group interaction, Model 5 we also control for group differences and Model 6 fits a model with a three-way interaction between baseline level, time, and group. The table presents estimates of fixed effects, random effects, and model fit (AIC). Significance levels: * *P*<0.05, ** *P*<0.01, *** *P*<0.001.

|  | **Model 1** | | **Model 2** | | | **Model 3** | | | **Model 4** | | | **Model 5** | | | **Model 6** | |
| --- | --- | --- | --- | --- | --- | --- | --- | --- | --- | --- | --- | --- | --- | --- | --- | --- |
| *Predictors* | *Estimates* |  | *Estimates* | |  | *Estimates* | |  | *Estimates* | |  | *Estimates* | |  | *Estimates* |  |
| (Intercept) | 0.12 ^**^ |  | | 0.04 |  | | 0.04 |  | | 0.04 |  | | 0.07 |  | 0.01 |  |
| Time |  |  | | 0.19 ^***^ |  | | 0.19 ^***^ |  | | 0.09 ^**^ |  | | 0.09 ^*^ |  | 0.10 ^**^ |  |
| Time * Intervention |  |  | |  |  | |  |  | | 0.26 ^***^ |  | | 0.28 ^***^ |  | 0.25 ^***^ |  |
| Intervention |  |  | |  |  | |  |  | |  |  | | -0.07 |  | -0.01 |  |
| Baseline Global wellbeing |  |  | |  |  | |  |  | |  |  | |  |  | 0.98 ^***^ |  |
| Time * Baseline Global wellbeing |  |  | |  |  | |  |  | |  |  | |  |  | -0.17 ^***^ |  |
| Intervention * Baseline Global wellbeing |  |  | |  |  | |  |  | |  |  | |  |  | 0.01 |  |
| Time * Intervention *baseline Global wellbeing |  |  | |  |  | |  |  | |  |  | |  |  | -0.02 |  |
| **Random Effects** | | | | | | | | | | | | | | | | |
| σ^2^ | 0.18 | | 0.17 | | | 0.14 | | | 0.15 | | | 0.15 | | | 0.07 | |
| τ_00_ | 0.86 | | 0.86 | | | 0.87 | | | 0.87 | | | 0.87 | | | 0.00 | |
| τ_11_ |  | |  | | | 0.12 | | | 0.10 | | | 0.10 | | | 0.35 | |
| ρ_01_ |  | |  | | | -0.06 | | | -0.05 | | | -0.05 | | | 1.00 | |
| AIC | 3408.114 | | 3364.617 | | | 3354.652 | | | 3338.576 | | | 3343.081 | | | 1359.609 | |
| ** p<0.05   ** p<0.01   *** p<0.001* | | | | | | | | | | | | | | | | |

**Table 2E**: Mixed model results for the effects of time, intervention, and their interaction in SCL. Model 1 includes a fixed and random intercept, Model 2 adds the fixed effect of time, Model 3 also incorporates a random slope for time, Model 4 adds a time by group interaction, Model 5 we also control for group differences and Model 6 fits a model with a three-way interaction between baseline level, time, and group. The table presents estimates of fixed effects, random effects, and model fit (AIC). Significance levels: * *P*<0.05, ** *P*<0.01, *** *P*<0.001.

|  | **Model 1** | **Model 2** | **Model 3** | **Model 4** | **Model 5** | **Model 6** |
| --- | --- | --- | --- | --- | --- | --- |
| *Predictors* | *Estimates* | *Estimates* | *Estimates* | *Estimates* | *Estimates* | *Estimates* |
| (Intercept) | -0.08 ^*^ | -0.01 | -0.01 | -0.01 | -0.05 | 0.01 |
| Time |  | -0.17 ^***^ | -0.17 ^***^ | -0.12 ^**^ | -0.11 ^*^ | -0.10 ^*^ |
| Time * Intervention |  |  |  | -0.14 ^*^ | -0.17 ^**^ | -0.18 ^**^ |
| Intervention |  |  |  |  | 0.09 | 0.01 |
| Baseline SCL |  |  |  |  |  | 0.98 ^***^ |
| Time * Baseline SCL |  |  |  |  |  | -0.26 ^***^ |
| Intervention * Baseline SCL |  |  |  |  |  | 0.01 |
| Time * Intervention * Baseline SCL |  |  |  |  |  | -0.19 ^**^ |
| **Random Effects** | | | | | | |
| σ^2^ | 0.25 | 0.25 | 0.23 | 0.23 | 0.23 | 0.11 |
| τ_00_ | 0.72 | 0.72 | 0.79 | 0.79 | 0.79 | 0.00 |
| τ_11_ |  |  | 0.08 | 0.07 | 0.07 | 0.37 |
| ρ_01_ |  |  | -0.35 | -0.34 | -0.34 | 1.00 |
| AIC | 3645.871 | 3623.911 | 3622.473 | 3622.862 | 3626.741 | 1925.811 |
| ** p<0.05   ** p<0.01   *** p<0.001* | | | | | | |
